# Cross-validation and sensitivity to change of EULAR ScleroID as a measure of function and impact of disease in patients with systemic sclerosis

**DOI:** 10.1136/rmdopen-2025-005999

**Published:** 2025-10-10

**Authors:** Seda Yurumez Colak, Stefano Di Donato, Riccardo Bixio, Lesley-Anne Bissell, Theresa Barnes, Muhammad Nisar, Vishal Kakkar, Chris Denton, Francesco Del Galdo

**Affiliations:** 1 Leeds Institute of Rheumatic and Musculoskeletal Medicine, University of Leeds Faculty of Medicine and Health, Leeds, UK; 2 Gulhane Training and Research Hospital, Ankara, Turkey; 3 Department of Rheumatology, Countess of Chester Hospital NHS Foundation Trust, Chester, UK; 4 Department of Rheumatology, Luton and Dunstable University Hospital NHS Foundation Trust, Luton, UK; 5 Department of Rheumatology, Royal Free London NHS Foundation Trust, London, England, UK; 6 NIHR Biomedical Research Centre, LTHT, Leeds, UK

**Keywords:** Systemic Sclerosis, Disease Activity, Patient Reported Outcome Measures

## Abstract

**Objective:**

To determine the distribution of the EULAR SSc Impact of Disease (ScleroID) and its domain questions in very early (Ve), limited (lc) and diffuse cutaneous (dc) subsets, its value in reflecting clinical severity, and to assess its sensitivity to change and minimal clinically important difference (MCID) in a 12-month interval.

**Methods:**

Patients with ScleroID questionnaires from the observational cohort STRIKE were included in the study. Changes (Δ) were calculated as the difference between 12-month follow-up and compared MCIDs of the other measures.

**Results:**

Data were available for 271 patients, 69 with Ve, 139 lc and 63 dc systemic sclerosis (SSc). Median (IQR) ScleroID scores were progressively higher in the 3 subsets with 2.1 (3.6) for VeSSc, 3.4 (4.4) for lcSSc and 4.7 (4) for dcSSc (p<0.001). ScleroID showed strong content validity against clinical measures. Patients with high disease activity had significantly higher ScleroID scores than low ones (p=0.003). Presence of digital ulcers, pulmonary disease or small intestinal bacterial overgrowth was all reflected in higher scores in their relative domains (p<0.005 for all). Accordingly, ScleroID scores and its relative domains showed high correlations with all other patient-reported outcomes (PROs) (p<0.05). Changes in ScleroID strongly correlated with changes in clinical measures and other PROs with specific thresholds identified for MCID changes in Health Assessment Questionnaire Disability Index, the University of California Los Angeles Scleroderma Clinical Trials Consortium gastrointestinal tract 2.0 and Cochin Hand Function Scale.

**Conclusion:**

ScleroID demonstrates strong correlation with validated clinical measures and responsiveness to changes in standard of care, supporting its use in both clinical practice and trials. ScleroID captures the multidimensional burden of SSc regardless of disease subsets.

**WHAT IS ALREADY KNOWN ON THIS TOPIC**

- Multiple patient-reported outcomes (PROs) have been developed to capture specific aspects of patient burden in systemic sclerosis (SSc).
- The EULAR SSc Impact of Disease (ScleroID) questionnaire is a multidomain PRO capturing the most important domains of SSc as prioritised by patients and doctors.

**WHAT THIS STUDY ADDS**

- ScleroID and its sub-items show good correlation especially with related clinical measures and PROs.
- Across the SSc disease subsets, ScleroID shows a good impact at the baseline and 12-month follow-up time and with MCID cut-offs defined for Health Assessment Questionnaire Disability Index, University of California Los Angeles Scleroderma Clinical Trials Consortium gastrointestinal tract 2.0 and Cochin Hand Function Scale.
- ScleroID correlation with validated clinical measures and its sensitivity to change in standard of care setting supports its use as a key composite PRO in clinical practice and clinical trials.

**HOW THIS STUDY MIGHT AFFECT RESEARCH, PRACTICE OR POLICY**

- This study contributes to the use of EULAR ScleroID questionnaire as a comprehensive, easy to perform PRO able to capture the overall impact of SSc on patients across the distinct disease subsets.

## Introduction

 Systemic sclerosis (SSc) is a multisystem chronic disease with skin and internal organ manifestations, associated with significant decrease in quality of life and the highest morbidity and mortality across rheumatic diseases. Patient-reported outcome (PROs) measures are central to evaluating the impact of disease on patients, including burden of specific manifestations, treatment response and overall function.^1 2^ However, most of the PROs used for SSc have been developed for other musculoskeletal conditions, for example, the Health Assessment Questionnaire Disability Index (HAQ-DI); or purely for a specified manifestation of the disease, such as the University of California Los Angeles Scleroderma Clinical Trials Consortium gastrointestinal tract 2.0 (UCLA GIT 2.0) or the Raynaud’s condition score.^2^,^6^ The identification of a comprehensive PRO that could capture overall disease burden has been prioritised in the research agenda of the recent EULAR recommendations for treatment of SSc.^7^ The EULAR Systemic Sclerosis Impact of Disease (ScleroID) questionnaire is a PRO measure developed with wide patient engagement, aiming to capture the overall impact of the disease, while reporting on ten domains prioritised through validated data driven methodology.^8^ The 10 domains include: Raynaud’s phenomenon; hand function; digital ulceration (DU); both upper and lower gastrointestinal symptoms; dyspnoea; pain; fatigue; and effects on body mobility, life choices and activities.

Previous studies have correlated the ScleroID total score with many other PRO measures commonly used in SSc, including Patient Global Assessment, scleroderma HAQ (sHAQ), Short-form health survey 36 (SF-36), Cochin Hand Function Scale (CHFS), UCLA GIT 2.0, skin thickness and revised EUSTAR disease activity index.^8 9^ However, there is no data related to the sensitivity to change of the ScleroID over time, neither how changes in ScleroID relate to Minimal Clinically Important Differences (MCID) in clinical and PRO domains.

Several SSc domains, such as CHFS, skin involvement (modified Rodnan Skin Score (mRSS)), lung function and HAQ, are known to change dynamically over time and are targeted in clinical trials. Further, composite trial endpoints such as Composite Response Index in Systemic Sclerosis already incorporate PROs to assess treatment efficacy. On these premises, we hypothesised that the ScleroID, specifically designed for SSc and inclusive of multiple disease-relevant domains, may not only correlate with other PROs and clinical outcomes, but also serve as a single, comprehensive tool to detect meaningful clinical changes over time across all subsets of SSc.^410^,^14^

For this purpose, we evaluated the distribution of ScleroID scores within different subsets of SSc including very early (Ve), limited cutaneous (lc) and diffuse cutaneous (dc) SSc. Within these subsets, we evaluated the performance of ScleroID in capturing the impact of disease from other PROs and clinical measures. Most importantly, we evaluated the ScleroID sensitivity to changes according to published MCIDs of HAQ, CHFS and UCLA GIT 2.0.

## Material and methods

### Patients and study design

This was a retrospective analysis of a prospective observational study. Participants who had completed EULAR ScleroID questionnaires in the prospective observational Stratification for Risk of Progression in Systemic Sclerosis (STRIKE) cohort between 1 January 2022 and 1 February 2024 were included in this analysis. Patients in STRIKE included male and female adults with either a diagnosis of SSc fulfilling the 2013 American College of Rheumatology–European Alliance of Associations for Rheumatology (EULAR) SSc classification criteria^15^ or with Raynaud’s phenomenon fulfilling very early diagnosis of SSc criteria.^16^ Patients who fulfilled the EULAR SSc criteria were further classified as limited or diffuse SSc according to the LeRoy classification.^17^ Patients who had chronic pain syndromes or were meeting diagnostic criteria for other connective tissue diseases were excluded. Patients with available 12-month follow-up questionnaires were identified as the longitudinal cohort. A study was conducted within the protocol of STRIKE approved by NHS Health Research Authority (REC 15/NE/0211, IRAS ID 178638). Written informed consent form was obtained from all participants.

### Data collection, clinical measurements and PROs

Demographic and clinical data including age, gender, disease subtype, disease duration, presence of autoantibodies and clinical manifestations such as digital ulcers, calcinosis, mRSS, gastro-oesophageal reflux disease, small intestine bacterial overgrowth (SIBO), interstitial lung disease (ILD) and pulmonary arterial hypertension (PAH) were collected from the STRIKE database. Predicted values of forced vital capacity (FVC), diffusion capacity of carbon monoxide (DLco) and mRSS at the visit dates were also collected from the STRIKE database. Other PROs recorded included: UCLA GIT 2.0, CHFS, Borg rating of perceived exertion scale (BORG), HAQ-DI and sHAQ.^4^,^618^ The PROs were calculated as reported in previous studies.^4^,^68^

The modified version of the EUSTAR Disease Activity Index (mDAI)^13^ was used to evaluate the disease activity in patients with SSc, with scores <2.5 representing inactive/moderately active disease and ≥2.5 active/very active disease (maximum score 8.5).^19^

ScleroID has ten sub-items: Raynaud’s phenomenon, hand function, upper gastrointestinal, pain, fatigue, lower gastrointestinal, life choices and activities limitation, body mobility, dyspnoea and digital ulcer. Each domain is multiplied by its weighted value, and the sum of the values gives the total ScleroID score as described previously.^8^

### Longitudinal analysis of sensitivity to change

In the longitudinal cohort, ScleroID scores, clinical findings and other PROs were assessed at 12-month follow-up visits. The changes (Δ) of scores were calculated as the difference between the 12-month follow-up and baseline scores for each questionnaire and the clinical measurements.

To analyse the sensitivity to change in ScleroID scores, we anchored changes on previously published MCID thresholds for HAQ-DI, CHFS and UCLA GIT 2.0.^4 19 20^ Briefly, the MCIDs for HAQ-DI were accepted as a decrease by 0.2 points for improvement and an increase by 0.2 points for worsening.^20^ For CHFS, patients scoring more than patient acceptable symptom state of 26 points were considered for MCID values of worsening if they had an increase of 21.6% or absolute 1.4 points increase. Improved CHFS was a decrease of 13.1% or 3.4 point absolute decrease.^4 20^ The MCID for worsening UCLA GIT 2.0 total score was 0.12 points, and 0.18 points for improvement.^21^

### Statistical analysis

IBM SPSS for Mac (V.29.0) was used for the statistical analyses. Categorical variables were defined as counts and percentages. Mean (± SD) or median (lower and upper quartile or IQR) values were used for continuous variables according to data distribution. The χ2, Fisher’s exact test, Mann-Whitney U, Kruskal-Wallis or ne-way analysis of variance was used to compare the differences between subgroups as appropriate. Wilcoxon ranked test was used to assess differences between baseline and follow-up scores. Spearman’s rank correlation test was used to analyse the correlations between ScleroID scores and other PROs and clinical measurements. The correlations were analysed for baseline, follow-up and the differences over time. Significance of correlations and exploratory group comparisons across disease phenotypes was adjusted with Benjamini-Hochberg False Discovery Rate (q value) to avoid type II error. A p value <0.05 was considered statistically significant.

## Results

### Patients’ characteristics

**Figure 1:**
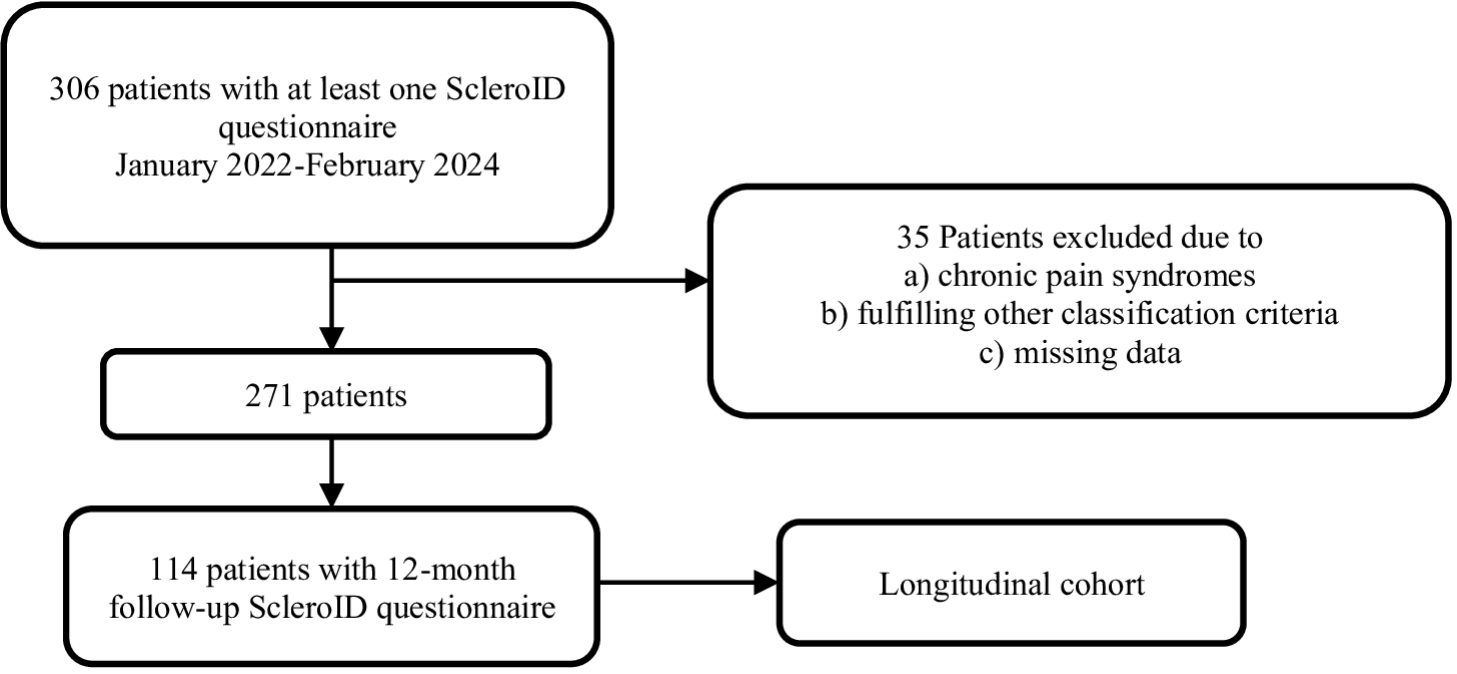
Patient selection and study group. ScleroID, EULAR SSc Impact of Disease.

Three hundred and six patients had ScleroID available in our study time frame. Of these, 35 patients were excluded due to concurrent diagnosis of other chronic pain syndromes or meeting diagnostic criteria for other connective tissue diseases (figure 1). The 271 remaining patients (245 female) had a mean age of 54.8±13.9 years. The baseline demographic and clinical features of the participants are shown in table 1. Sixty-nine patients had VeSSc, 139 patients had lcSSc and 63 patients had dcSSc. The patients with VeSSc were significantly younger than patients with lcSSc and dcSSc. Other significant differences included higher frequency of ILD, SIBO and tendon friction rub in the dcSSc subset.

**Table 1:** Baseline characteristics of the study group

Variable	Overall patients (n=271)	VeSSc (n=69)	Limited SSc (n=139)	Diffuse SSc (n=63)	Adjusted p value*
Age in years, mean±SD	54.8±13.9	47.2±12.7	59.1±13.3	53.6±12.7	**0.002**
Gender: female, n (%)	245 (90.4)	64 (92.3)	127 (91.4)	54 (85.7)	0.636
Gender: male, n (%)	26 (9.6)	5 (7.7)	12 (8.6)	9 (14.3)	0.636
Disease duration in months, median (IQR)	–	–	94 (141)	80 (95)	0.57
mRSS, median (IQR)	–	–	2 (3)	7 (10)	**0.002**
Forced vital capacity, % predicted	99.3±22.7	106.1±15.6	103.6±22	84.8±22	**0.002**
Diffusion capacity of carbon monoxide, % predicted	71.6±18.2	86.9±17.3	70.5±15.2	63.5±19.3	**0.002**
GERD, n (%)	152 (56.1)	20 (29.1)	93 (66.9)	39 (61.9)	**0.002**
Interstitial lung disease, n (%)	72 (35.6)	–	31 (22.3)	41 (65.1)	**0.002**
Digital ulcer, n (%)	63 (31.2)	–	38 (27.3)	25 (39.7)	0.15
Calcinosis, n (%)	59 (29.2)	–	43 (30.9)	16 (25.4)	0.567
SIBO, n (%)	34 (12.5)	1 (0.5)	18 (12.9)	15 (23.8)	0.106
PAH, n (%)	19 (9.4)	–	11 (7.9)	8 (12.7)	0.393
Tendon friction rub, n (%)	13 (6.4)	–	5 (3.6)	8 (12.7)	**0.04**
Anti-nuclear antibody, n (%)	261 (96.3)	69 (100)	132 (95.1)	60 (95.2)	0.208
Anti-centromere, n (%)	94 (47.2)	34 (49.3)	91 (65.5)	3 (4.8)	**0.002**
Anti Scl-70, n (%)	55 (25.5)	14 (20.3)	19 (13.7)	36 (57.1)	**0.002**
Anti RNP, n (%)	21 (8.9)	3 (1.5)	6 (4.3)	15 (23.8)	**0.002**

* Analysis of variance, Mann-Witney U test, χ² or Fisher’s exact test as appropriate based on data and number of groups.

GERD, gastro-oeosophageal reflux disease; mRSS, modified Rodnan Skin Score; PAH, pulmonary arterial hypertension; RNP, ribonucleoprotein; SIBO, small intestinal bacterial overgrowth; VeSSc, very early systemic clerosis.

### ScleroID across SSc subsets

**Figure 2:**
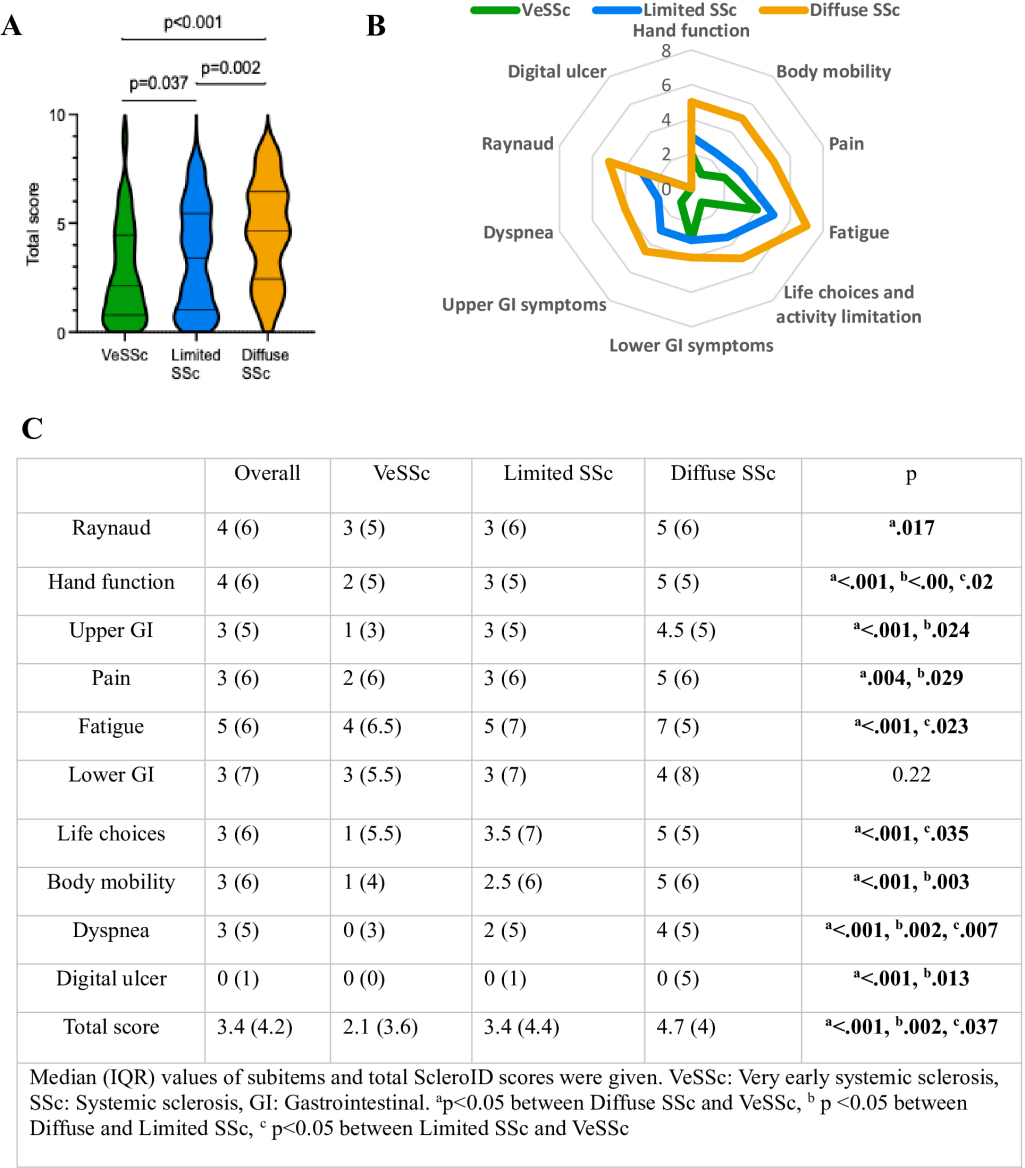
(**A**) The violin plots of the median and 25-75th quartiles ScleroID total scores of each disease subset. (**B**) The radar graph of the median scores of each sub-item according to disease subsets with the colour chosen. (**C**) The table of baseline median (IQR) values of overall patients and disease subsets. Mann-Whitney U and Kruskal-Wallis tests were used for statistical analysis.

The median (IQR) ScleroID in the whole study population was 3.4 (4.2). There were no significant differences in ScleroID and sub-item scores according to age, sex and presence of autoantibodies. ScleroID sub-items and total scores of the patients according to disease subset and overall study group are presented in figure 2. The median (IQR) ScleroID scores were significantly different among disease subsets with 4.7 (4) in dcSSc, 3.4 (4.4) in lcSSc and 2.1 (3.6) in VeSSc patients (p<0.05). In terms of sub-item scores, only lower GI scores were similar between all disease subsets. Patients with dcSSc had significantly higher scores in all other domains compared with VeSSc and in hand function, upper GI, pain, body mobility, dyspnoea and digital ulcer scores compared with lcSSc. LcSSc patients had significantly higher hand function, fatigue, life choices and dyspnoea scores than VeSSc patients. A schematic representation of subdomain distribution across disease subsets is summarised in the radar plots of figure 2B.

### Impact of disease manifestations on ScleroID

Next, we aimed to capture the impact of specific disease manifestations on ScleroID scores, independently of cutaneous subset. The highest ScleroID scores were recorded in patients with PAH with median (IQR) ScleroID total score of 5.8 (6.1) followed by 5.4 (4.1) in DU, 5.2 (3.8) in SIBO, 4.4 (3.8) in ILD and 4.1 (4.1) in calcinosis. Distribution of the ScleroID domains also reflected clinical manifestations with, for instance, the highest median (IQR) Raynaud score of 7 (5) in patients with DU, highest dyspnoea median of 6 (5) in patients with PAH or the highest median lower GI of 7 (4) in patients with SIBO. The distribution of scores according to presence or absence of distinct clinical manifestations is shown in table 2.

**Table 2:** The ScleroID scores according to the clinical manifestations

	ILD			PAH			Digital ulcer			Calcinosis			SIBO		
	Present	Absent	Adjusted P value	Present	Absent	Adjusted P value	Present	Absent	Adjusted P value	Present	Absent	Adjusted P value	Present	Absent	Adjusted P value
Raynaud	4 (5)	3 (6)	0.58	6 (6)	4 (6)	0.49	7 (5)	3 (5)	**0.002**	3.5 (6)	4 (6)	0.67	6 (5)	3 (6)	**0.041**
Hand function	5 (5)	3 (5)	0.44	7 (7)	4 (5)	0.39	6 (5)	3 (5)	**0.002**	4.5 (5)	4 (6)	0.33	6 (4)	4 (5)	**0.015**
Upper GI	4 (6)	3 (5)	0.61	5.5 (8)	3 (5)	0.39	5 (7)	3 (5)	**0.022**	4 (5)	3 (5)	0.45	7 (5)	3 (5)	**0.002**
Pain	4 (6)	3 (7)	0.92	5.5 (7)	3 (6)	0.52	5.5 (6)	3 (6)	**0.002**	4 (6)	3 (6)	0.45	5 (6)	3 (6)	**0.018**
Fatigue	6 (6)	5 (7)	0.52	8 (6)	5 (6)	0.14	7 (5)	5 (6)	**0.003**	6 (6)	5 (6)	0.45	7 (6)	5 (6)	0.23
Lower GI	4 (6)	2.5 (7)	0.58	6 (8)	3 (7)	0.39	5 (7)	3 (7)	0.06	3 (7)	3 (7)	0.44	7 (4)	2.5 (6)	**0.002**
Life choices	5 (6)	3 (7)	0.44	6 (6)	4 (7)	0.31	6 (6)	3 (6)	**0.002**	5 (7)	3 (6)	0.33	6 (5)	3 (6)	**0.003**
Body mobility	5 (5)	2 (6)	**0.038**	7.5 (7)	3 (6)	0.06	5 (6)	3 (6)	**0.003**	4 (7)	3 (6)	0.35	6 (6)	3 (6)	**0.031**
Dyspnoea	4 (5)	1.5 (4)	**0.011**	6 (5)	2 (5)	**0.011**	4 (7)	2 (5)	**0.007**	2 (5)	3 (5)	0.78	4 (7)	2 (5)	0.21
Digital ulcer	0 (3)	0 (1)	0.44	0 (7)	0 (2)	0.40	4 (7)	0 (0)	**0.002**	0.5 (4)	0 (1)	0.33	0 (4)	0 (2)	0.47
Total score	4.4 (3.8)	3.3 (4.8)	0.29	5.8 (6.1)	3.7 (4.1)	0.18	5.4 (4.1)	3.3 (3.9)	**0.002**	4.1 (4.1)	3.8 (4.4)	0.15	5.2 (3.8)	3.4 (4.1)	**0.002**

GI, gastrointestinal; ILD, interstitial lung disease; PAH, pulmonary arterial hypertension; SIBO, small intestine bacterial overgrowth.

It was also important to note that patients with ILD had higher scores for dyspnoea and body mobility than patients without ILD (adjusted p=0.011 and p=0.038, respectively), but there was no difference in the total score (p>0.05). Patients with PAH had higher scores of dyspnoea than patients without PAH (p=0.011), though no difference in the total score (p>0.05). Patients with PAH also had higher scores of fatigue and body mobility than patients without PAH, and the unadjusted p value were <0.05; however, the association did not remain statistically significant after multiple comparisons. Patients with DUs had higher total ScleroID and sub-item scores except for lower GI score than patients without DUs (p<0.05). The presence of calcinosis was not related to higher ScleroID sub-item or total scores (adjusted p>0.05). Patients who had SIBO had higher total, upper GI and lower GI scores than patients without SIBO (adjusted p<0.005). (table 2, online supplemental figure 1).

### ScleroID and modified disease activity score

The median (IQR) mDAI was 1.2 (2.3) in the study group. There were 144 (71.3%) patients with inactive/moderately active disease and 58 (28.7%) with active/very active disease. The median (IQR) total ScleroID score was 4.7 (4.5) in active/very active patients, and it was 3.6 (4.3) in inactive/moderately active patients (p=0.003). Higher median (IQR) scores in patients with high mDAI were driven by worse hand function (5 (6) vs 3 (5), p=0.004), life choices (5 (7) vs 3 (6), p=0.02), body mobility (5 (7) vs 2.5 (6), p=0.009) and dyspnoea (4 (5) vs 2 (5), p<0.001), sub-item scores (online supplemental figure 2).

### Correlation with clinical measurements and PROs

The correlation of ScleroID total score and clinical measurements and PROs was published before (7,8). Consistent with published findings, in our cohort ScleroID total score was well correlated with clinical findings including mRSS, FVC, and DLco, mDAI and PROs (table 3). ScleroID total score showed excellent correlation with HAQ-DI scores (Rho=0.76, p<0.001), sHAQ (Rho=0.73, p<0.001) and visual analogue scale (VAS) disease severity (Rho=0.69, p<0.001). In general, the PROs correlated better with their related sub-item scores than ScleroID total score. Indeed, the correlation rate between BORG and dyspnoea score was 0.89 (adjusted p=0.001), 0.69 (adjusted p=0.0012) between CHFS and hand function and 0.72 (adjusted p=0.0012) between UCLA GIT 2.0 Reflux and upper GI. Lower GI was better correlated with other subdomains and total score of UCLA GIT 2.0 than ScleroID total score (table 3).

**Table 3:** Correlations between ScleroID scores and clinical and PRO measurements

	Raynaud		Hand function		Upper GI		Pain		Fatigue		Lower GI		Life choices		Body mobility		Dyspnoea		Digital ulcer		Total score	
	Rho	Adj. P	Rho	Adj. P	Rho	Adj. P	Rho	Adj. P	Rho	Adj. P	Rho	Adj. P	Rho	Adj. P	Rho	Adj. P	Rho	Adj. P	Rho	Adj. P	Rho	Adj. P
BORG	0.34	**0.0012**	0.50	**0.0012**	0.60	**0.0012**	0.50	**0.0013**	0.57	**0.0012**	0.53	**0.0012**	0.54	**0.0013**	0.58	**0.0012**	0.89	**0.001**	0.35	**0.0017**	0.67	**<0.001**
CHFS	0.46	**0.0012**	0.69	**0.0012**	0.55	**0.0012**	0.59	**0.0013**	0.57	**0.0012**	0.43	**0.0012**	0.53	**0.0013**	0.62	**0.0012**	0.45	**0.001**	0.39	**0.0017**	0.68	**<0.001**
GIT reflux	0.35	**0.0012**	0.49	**0.0012**	0.72	**0.0012**	0.50	**0.0013**	0.53	**0.0012**	0.52	**0.0012**	0.50	**0.0013**	0.49	**0.0012**	0.46	**0.001**	0.22	**0.0017**	0.62	**<0.001**
GIT distension	0.25	**0.0012**	0.33	**0.0012**	0.48	**0.0012**	0.42	**0.0013**	0.48	**0.0012**	0.64	**0.0012**	0.42	**0.0013**	0.46	**0.0012**	0.39	**0.001**	0.13	0.058	0.52	**<0.001**
GIT soilage	0.16	**0.012**	0.24	**0.0012**	0.30	**0.0012**	0.21	**0.0023**	0.35	**0.0012**	0.48	**0.0012**	0.29	**0.0013**	0.25	**0.0012**	0.34	**0.001**	0.16	**0.012**	0.33	**<0.001**
GIT diarrhoea	0.12	0.069	0.19	**0.0034**	0.33	**0.0012**	0.22	**0.0013**	0.25	**0.0012**	0.51	**0.0012**	0.27	**0.0023**	0.20	**0.0093**	0.24	**0.001**	0.12	0.077	0.31	**<0.001**
GIT SF	0.36	**0.0012**	0.45	**0.0012**	0.51	**0.0012**	0.47	**0.0013**	0.56	**0.0012**	0.68	**0.0012**	0.47	**0.0013**	0.48	**0.0012**	0.45	**0.001**	0.21	**0.0017**	0.59	**<0.001**
GIT EWB	0.33	**0.0012**	0.35	**0.0012**	0.48	**0.0012**	0.36	**0.0013**	0.47	**0.0012**	0.72	**0.0012**	0.39	**0.0013**	0.41	**0.0012**	0.42	**0.001**	0.19	**0.0044**	0.53	**<0.001**
GIT constipation	0.18	**0.005**	0.18	**0.005**	0.29	**0.0012**	0.24	**0.0013**	0.23	**0.0012**	0.33	**0.0012**	0.27	**0.0023**	0.29	**0.0012**	0.22	**0.001**	0.10	0.15	0.29	**<0.001**
GIT total	0.30	**0.0012**	0.40	**0.0012**	0.56	**0.0012**	0.44	**0.0013**	0.52	**0.0012**	0.73	**0.0012**	0.45	**0.0013**	0.46	**0.0012**	0.46	**0.001**	0.22	**0.0017**	0.58	**<0.001**
HAQ	0.47	**0.0012**	0.67	**0.0012**	0.60	**0.0012**	0.62	**0.0013**	0.66	**0.0012**	0.51	**0.0012**	0.68	**0.0013**	0.76	**0.0012**	0.53	**0.001**	0.34	**0.0017**	0.76	**<0.001**
VAS severity	0.46	**0.0012**	0.58	**0.0012**	0.54	**0.0012**	0.61	**0.0013**	0.59	**0.0012**	0.47	**0.0012**	0.65	**0.0013**	0.67	**0.0012**	0.46	**0.001**	0.31	**0.0017**	0.69	**<0.001**
VAS pain	0.43	**0.0012**	0.54	**0.0012**	0.49	**0.0012**	0.70	**0.0013**	0.61	**0.0012**	0.48	**0.0012**	0.57	**0.0013**	0.61	**0.0012**	0.39	**0.001**	0.25	**0.0017**	0.66	**<0.001**
VAS articular	0.36	**0.0012**	0.54	**0.0012**	0.48	**0.0012**	0.59	**0.0013**	0.61	**0.0012**	0.45	**0.0012**	0.53	**0.0013**	0.60	**0.0012**	0.37	**0.001**	0.21	**0.0044**	0.62	**<0.001**
VAS intestinal	0.32	**0.0012**	0.42	**0.0012**	0.62	**0.0012**	0.49	**0.0013**	0.53	**0.0012**	0.70	**0.0012**	0.54	**0.0013**	0.50	**0.0012**	0.49	**0.001**	0.17	**0.0096**	0.61	**<0.001**
VAS dyspnoea	0.38	**0.0012**	0.49	**0.0012**	0.54	**0.0012**	0.51	**0.0013**	0.59	**0.0012**	0.52	**0.0012**	0.54	**0.0013**	0.59	**0.0012**	0.86	**0.001**	0.29	**0.0017**	0.67	**<0.001**
VAS Raynaud	0.79	**0.0012**	0.62	**0.0012**	0.47	**0.0012**	0.53	**0.0013**	0.42	**0.0012**	0.38	**0.0012**	0.46	**0.0013**	0.44	**0.0012**	0.43	**0.001**	0.33	**0.0017**	0.64	**<0.001**
VAS DU	0.32	**0.0012**	0.41	**0.0012**	0.23	**0.0012**	0.33	**0.0013**	0.22	**0.0012**	0.13	**0.045**	0.29	**0.0013**	0.31	**0.0012**	0.32	**0.001**	0.71	**0.0017**	0.39	**<0.001**
sHAQ	0.43	**0.0012**	0.59	**0.0012**	0.54	**0.0012**	0.64	**0.0013**	0.67	**0.0012**	0.54	**0.0012**	0.66	**0.0013**	0.67	**0.0012**	0.53	**0.001**	0.31	**0.0017**	0.73	**<0.001**
FVC	−0.14	0.069	−0.17	**0.033**	−0.23	**0.003**	−0.11	0.18	−0.18	**0.022**	−0.07	0.39	−0.16	**0.033**	−0.23	**0.0035**	−0.41	**0.001**	−0.12	0.13	−0.24	**0.002**
DLco	−0.11	0.16	−0.19	**0.021**	−0.17	**0.04**	−0.06	0.44	−0.10	0.21	−0.05	0.55	−0.14	0.08	−0.23	**0.0045**	−0.39	**0.001**	−0.21	**0.010**	0.21	**0.008**
mRSS	0.11	0.08	0.29	**0.0012**	0.17	**0.005**	0.12	0.064	0.10	0.10	0.09	0.16	0.13	**0.043**	0.16	**0.0075**	0.19	**0.002**	0.32	**0.0017**	0.20	**<0.001**
mDAI	0.1	0.17	0.23	**0.0012**	0.15	**0.04**	0.14	0.055	0.11	0.15	0.17	**0.023**	0.14	0.052	0.17	**0.011**	0.31	**0.001**	0.32	**0.0017**	0.23	**0.001**

CHFS, Cochin Hand Function Score; DLco, diffusion capacity of carbon monoxide; EWB, emotional well-being; FVC, forced vital capacity; GI, gastrointestinal; GIT, the University of California Los Angeles Scleroderma Clinical Trials Consortium gastrointestinal tract 2.0; HAQ, Health Assessment Questionnaire; mDAI, modified disease activity score; mRSS, Modified Rodnan Skin score; SF, social functioning; sHAQ, scleroderma HAQ; VAS, visual analogue scale.

### Longitudinal cohort analysis

**Figure 3:**
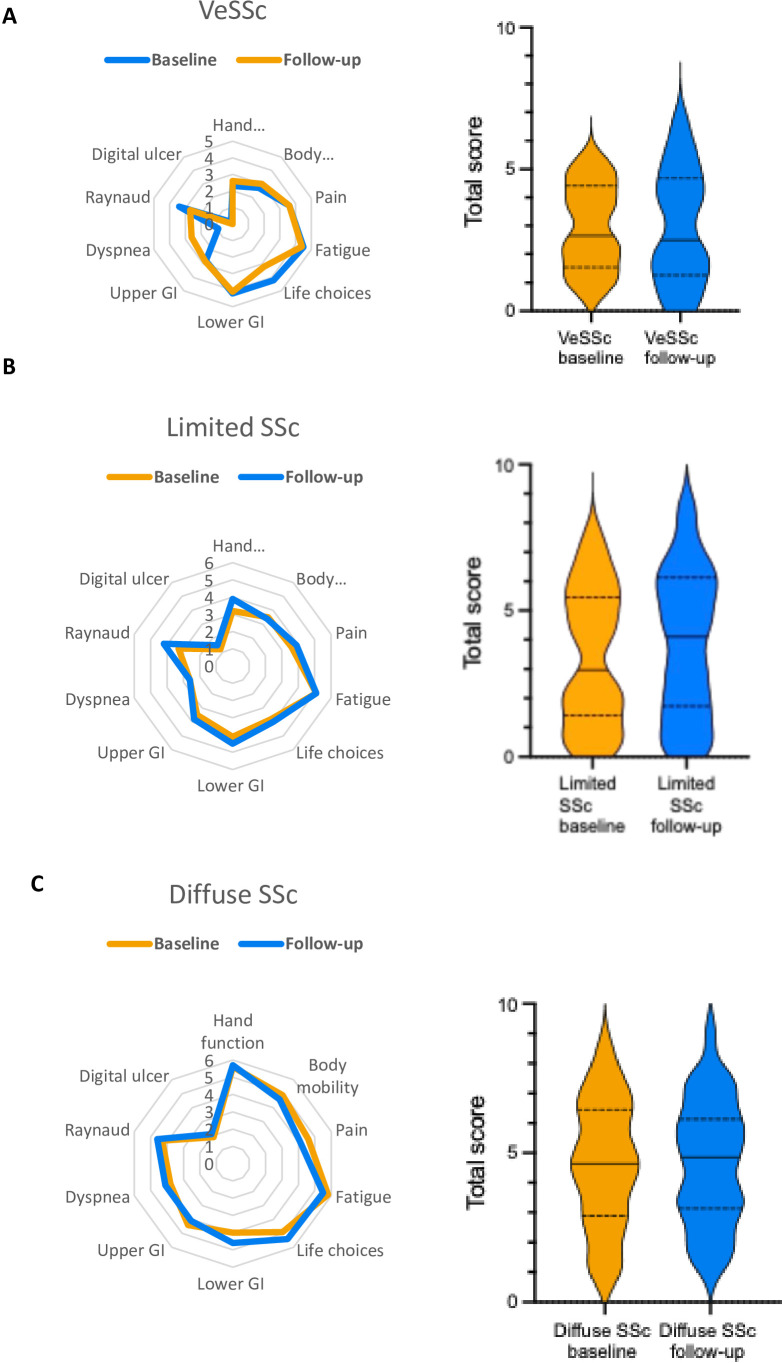
(**A**) The radar graphs of ScleroID subdomains and the violin plots of ScleroID total score of VeSSc patients at baseline and follow-up. (**B**) The radar graph of ScleroID subdomains and the violin plots of ScleroID total score of Limited SSc patients at baseline and follow-up. (**C**) The radar graph of ScleroID subdomains and the violin plots of ScleroID total score of diffuse SSc patients at baseline and follow-up. GI, gastrointestinal; ScleroID, EULAR SSc Impact of Disease; VeSSc, very early systemic sclerosis.

The 12-month ScleroID scores were available for 97 patients with SSc and 17 with VeSSc. The patients’ characteristics and the differences are shown in online supplemental table 1. VeSSc patients were younger than SSc patients and had higher predicted DLCO values, as expected. VeSSc patients in the longitudinal group had less anti-centromere antibody positivity and more scl-70 antibody positivity compared with the other VeSSc patients (p=0.02 and p=0.03, respectively). The clinical manifestations were similar between longitudinal and cross-sectional patients in the SSc group. ScleroID was relatively stable over time at group level, consistent with the observational nature of the cohort. Importantly, no ScleroID domains showed spontaneous improvement in 12 months, whereas most domains showed numerical worsening (figure 3).

The correlation between ScleroID scores and clinical findings and other PROs is given in table 3. Overall, there was a consistent, significant correlation between the change in ScleroID sub-items and the changes in the corresponding PROs. The strongest correlations were found between changes in the impact on life choices and body mobility with changes in HAQ-DI. There were also significant strongly positive correlations between the ΔBORG and Δdyspnoea domain of the ScleroID, as well as ΔCHFS with changes in the Raynaud’s and hand function ScleroID sub-items. Similarly, there were positive correlations between ΔUCLA GIT 2.0 scores and ΔGI lower and upper domains (online supplemental figure 3).

Altogether, these data confirmed the sensitivity to change of ScleroID and its domains in measuring the impact of SSc on quality of life, fatigue, functional status and symptoms related to gastrointestinal, skin and pulmonary involvements in the patients.

Next, we analysed the changes in ScleroID domains of patients meeting improving or worsening MCID in HAQ-DI, CHFS and UCLA GIT2.0 to define their sensitivity to changes and relative thresholds.

**Figure 4:**
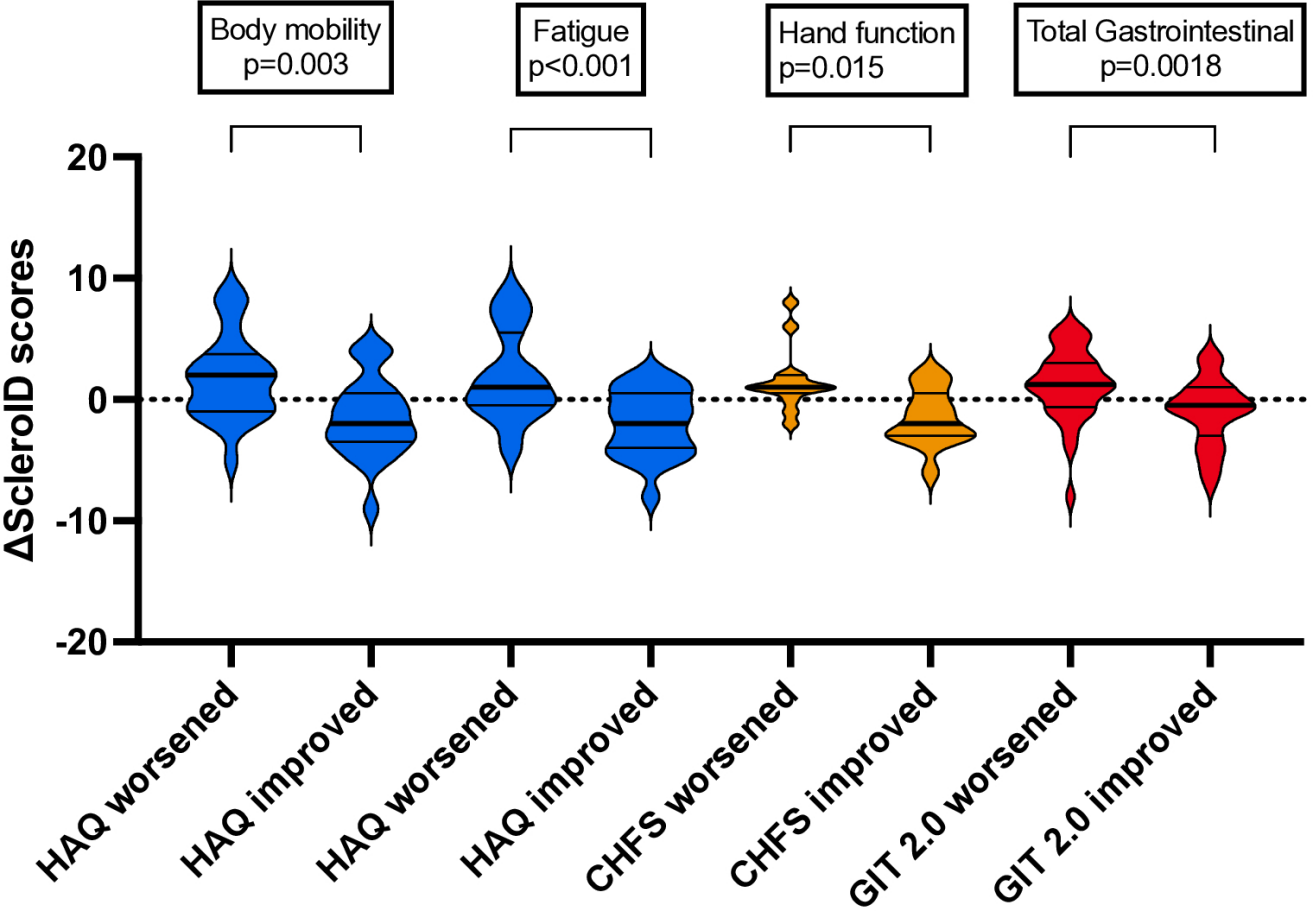
The improved and worsened patients according to HAQ, UCLA GIT 2.0 and CHFS and the changes of related ScleroID scores. Total gastrointestinal score indicates the average score of upper and lower gastrointestinal ScleroID sub-items. CHFS, Cochin Hand Function Scale; GIT 2.0, University of California Los Angeles Scleroderma Clinical Trials Consortium gastrointestinal tract 2.0; HAQ, Health Assessment Questionnaire; ScleroID, ScleroID, EULAR SSc Impact of Disease.

Twenty-five patients met MCID for worsening HAQ-DI and 22 patients for improvement. MCID worsening for HAQ-DI corresponded to 2 points worsening in body motility and life choices, with reciprocal changes in MCID improvers (figure 4A). There were also significant changes in fatigue, dyspnoea and total scores (online supplemental table 2).

Thirty-four patients worsened and 27 patients improved according to UCLA GIT 2.0 MCID. MCID worsening in UCLA GIT 2.0 corresponded to 1.25 points worsening in total GI scores whereas improvers showed 0.5 points improvement (figure 4B).

Ten patients worsened, and 13 patients improved according to CHFS MCID levels. MCID worsening or improvement in CHFS corresponded to 1 point worsening and 2 points improvement in hand function domain, respectively (figure 4C).

The detailed scores of the patients who improved or worsened according to HAQ, UCLA GIT 2.0 and CHFS MCIDs are shown in online supplemental table 2 and online supplemental figure 4.

## Discussion

This is the first study that investigates ScleroID and its sub-items in patients with SSc and VeSSc both cross-sectionally and longitudinally. This study considers the potential of ScleroID to be used in clinical trials as well as in routine daily practice as a unique multi-dimensional, decision-making PRO measure in SSc and VeSSc.

ScleroID is easy to perform for patients and to assess by physicians. Here, we showed that it is also well correlated with the other most used PROs in SSc. In this sense, it may be argued that ScleroID could replace other more complex PROs, particularly in routine clinical management, and it could be explored as an end point in clinical trials aiming at minimising overall disease burden in SSc. First, we observed that ScleroID does reflect the progressively worse impact of disease extent across the VeSSc versus lcSSc versus dcSSc continuum, confirming its face validity and extending its value to very early disease.

In the previous study by Becker *et al*, HAQ, UCLA GIT 2.0, CHFS and SF-36 correlated with ScleroID total score, whereas there was no difference in the clinical findings apart from the 6-min walking test.^8^ Our study did not find a significant difference in ScleroID total scores based on the presence of ILD, PAH, calcinosis and tendon friction rubs; however, scores were higher in patients with DUs and in those with SIBO. On the contrary, we showed that ScleroID sub-item scores generally reflected SSc-specific organ manifestations in the patients. For example, patients with ILD and PAH had higher dyspnoea scores than patients without, indicating that, when analysing the patient impact of a single organ manifestation, ScleroID sub-item scores should be used rather than the total score. A dilution effect due to the multidomain and heterogeneous structure of ScleroID may play a role in this. This may not come as a surprise in a complex multi-organ disease such as SSc, in which patients learn very well how to recognise the impact of disease in a specific domain. In this sense, we felt that the visualisation of Sclero-ID as a radar plot is particularly effective in giving not just the total score but also the distribution of the scores, giving a very graphical image of the impact of SSc on patients’ lives.

In the recent study reported by Naggy *et al*, correlations between ScleroID total score and other PROs were shown.^9^ Correlations between ScleroID total score and VAS and sHAQ have also been shown,^8^ along with UCLA GIT 2.0 severity classes.^9^ However, the correlation of ScleroID sub-items with related PROs has not been shown before. The current study has demonstrated that ScleroID total score and sub-item scores correlated with many of the PROs which are widely used in patients with SSc, including the UCLA GIT 2.0 total score. For the first time, we have evaluated the change in total ScleroID as well as its sub-item scores over time and correlated this change with clinical findings and other PROs. We have shown that the total ScleroID scores remain largely stable over a 12-month period, across disease subsets. Differences in sub-item scores only occurred in limited cutaneous disease for Raynaud’s and hand function scores. Although the follow-up time was relatively short, this is consistent with the natural disease course of patients with lcSSc.^22^ Total ScleroID score changes also correlated with CHFS, UCLA GIT 2.0 and HAQ variations over the 12-month period. Change in ScleroID sub-item scores positively correlated with their relative counterpart PROs changes, for instance, the change in the BORG scale correlated with the change in dyspnoea score. This suggests that ScleroID total score and, importantly, its sub-items, are sensitive to change and able to reflect changes in the disease course. On the other hand, the gastrointestinal domain, for instance, was less strongly correlated with organ involvement. This may suggest either a reduced responsiveness of the domain to clinical change or a genuine lack of change over time in gastrointestinal symptoms. In our cohort, relative ScleroID domains rather than total score were able to discriminate between patients who met the MCID for HAQ, UCLA GIT 2.0 and CHFS. ScleroID hand function sub-item detected the estimate change for CHFS, gastrointestinal sub-items for UCLA GIT 2.0 and fatigue, body mobility and life choices for HAQ.

The ScleroID hand function sub-item was able to detect predictive change for CHFS, the ScleroID gastrointestinal sub-items were able to detect predictive change for UCLA GIT 2.0, and fatigue, body mobility and life choices were able to detect predictive change for HAQ.

Nonetheless, the short follow-up time, relatively limited sample size and no correction for treatment or disease duration are some important limitations to consider when detecting significant differences. Continuing on the limitations, the follow-up period was limited to 12 months, which may not be sufficient to fully capture the long-term trajectories of ScleroID scores and their associations with clinical parameters. Future studies with extended follow-up durations and larger sample sizes may provide a more comprehensive understanding of the longitudinal dynamics of ScleroID and its clinical relevance. Another limitation is the relatively small number of patients with VeSSc included in the longitudinal cohort and the slightly higher prevalence of Scl-70 positive patients in this group, which may be driven by a selection bias for loss to follow-up of ACA positive patients. This may limit the generalisability of the longitudinal findings in the VeSSc population.

Since the longitudinal VeSSc group showed different serological characteristics compared with the baseline group incidentally, a potential selection bias may be introduced. This may limit the generalisability of the longitudinal findings in the VeSSc population. The small number of patients in the MCID-defined improved or worsened groups is an important limitation that warrants caution in the interpretation of our findings. Therefore, independent validation in larger and more diverse cohorts is necessary to confirm these preliminary observations. While several associations remained significant after multiple comparisons, these findings should be interpreted cautiously given the exploratory design of the study. Independent validation in larger, hypothesis-driven cohorts is warranted.

A validated threshold for severity has not yet been established for ScleroID, which limits the interpretability of severity-based analyses.

Within the limitation of small sample size and retrospective analysis, the anchoring of ScleroID subdomains changes to HAQ-DI MCID may support the clinical meaningfulness of 2 points improvement in body mobility and life choices, similar to 2 points improvement in hand function anchored on CHFS MCID. Conversely, for GI domains, a smaller improvement (1 point) does equate to MCID improvement of UCLA GIT 2.0. Independent validation of these thresholds will be crucial to inform the adoption of ScleroID sub-item thresholds in clinical trials.

In conclusion, our study demonstrates the potential of the ScleroID as a feasible, reliable and clinically anchored tool to serve as a substitute for multiple PROs in the assessment of patients with SSc. The ScleroID is a comprehensive disease impact assessment for patients with SSc and could be extended to patients with VeSSc. Implementation within clinical practice would be preferred to multiple time-consuming questionnaires and shows promise for utility in clinical trials. Although ScleroID shows good correlation and responsiveness, further validation in interventional studies is needed to confirm its utility as a surrogate endpoint in clinical trials.

## Supplementary material

10.1136/rmdopen-2025-005999 online supplemental file 1

## Data Availability

Data are available upon reasonable request.
