# Supplementary material for: Cross-validation and sensitivity to change of EULAR ScleroID as a measure of function and impact of disease in patients with systemic sclerosis
Source: RMD Open. 2025 Oct 10;11(4):e005999. doi: 10.1136/rmdopen-2025-005999 (PMC12516988; doi:10.1136/rmdopen-2025-005999)
Supplement: online supplemental file 1 [file rmdopen-11-4-s001.pdf]

Supplementary Materials

Supplementary Table 1. Demographic and clinical characteristics of the longitudinal cohort and the differences with non-longitudinal patients

	Longitudinal cohort (n=114)			VeSSc			SSc		
	SSc (n=97)	VeSSc (n=17)	p	Longitudinal (n=17)	Cross-sectional (n=52)	p	Longitudinal (n=97)	Cross-sectional (n=105)	p
Age, years, mean±SD	56±14.1	46.8±13.6	**0.014**	46.8±13.6	47.3±12.5	0.901	56±14.1	58.6±12.5	0.180
Gender, n (%)									
Female	85 (87)	16 (94.1)	0.688	16 (94.1)	48 (92.3)	0.980	85 (87)	96 (91.4)	0.490
Male	12 (13)	1 (5.9)		1 (5.9)	4 (7.7)		12 (13)	9 (8.6)	
Disease duration, months, median (IQR)	94 (36-176)	-		-	-		94 (36-176)	90 (45-152)	0.9
Disease type, n (%)									
Limited	67 (69.1)	-					67 (69.1)	72 (68.6)	1
Diffuse	30 (30.9)						30 (30.9)	33 (31.4)	
mRSS, median (IQR)	3 (0-7)	-		-	-		3 (0-7)	2 (0-6)	0.16
FVC	99.3±20.7	109.2±18.2	0.198	109.2±18.2	104.9±14.8	0.573	99.3±20.7	96.5±26.4	0.476
DLco	69.1±16.4	89.7±13.7	**<0.001**	89.7±13.7	85.9±18.7	0.607	69.1±16.4	67.8±17.3	0.672
Manifestations, n (%)									
GERD	66 (68)	6 (35.3)	**0.011**	6 (35.3)	14 (26.9)	0.547	66 (68)	66 (62.9)	0.463
Digital ulcer	29 (29.9)	0		0	0		29 (29.9)	34 (32.4)	0.471
ILD	37 (38.1)	0		0	0		37 (38.1)	36 (34.3)	0.557
Calcinosis	29 (29.9)	0		0	0		29 (29.9)	33 (31.4)	0.878
SIBO	18 (18.6)	1 (5.9)	0.298	1 (5.9)	0	0.246	18 (18.6)	15 (14.3)	0.450
PAH	7 (7.2)	0		0	0		7 (7.2)	12 (11.4)	0.343
TFR	10 (10.3)	0		0	0		10 (10.3)	4 (3.8)	0.153

Autoantibodies, n (%)									
ANA	90 (92.8)	17 (100)	0.169	17 (100)	52 (100)	0.699	90 (92.8)	102 (97.1)	0.2
ACA	44 (45.4)	4 (23.5)	0.114	4 (23.5)	30 (57.7)	**0.024**	44 (45.4)	50 (47.6)	0.779
Anti Scl-70	29 (29.9)	7 (41.2)	0.401	7 (41.2)	7 (13.5)	**0.032**	29 (29.9)	26 (24.8)	0.433
Anti RNP	9 (9.3)	1 (5.9)	0.542	1 (5.9)	2 (3.8)	0.578	9 (9.3)	12 (11.4	0.652

SSc: Systemic sclerosis, VeSSc: Very early systemic sclerosis, mRSS: Modified Rodnan skin score, FVC: Forced vital capacity, DLco: Diffusion capacity of carbon monoxide, GERD: Gastroesophageal reflux disease, ILD: Interstitial lung disease, SIBO: Small bacterial overgrowth, PAH: Pulmonary arterial hypertension, TFR: Tendon friction rub, ANA: Antinuclear antibody, ACA: Anticentromere antibody, RNP: Ribonucleoprotein, IQR: Interquartile range, SD: Standard deviation

Supplementary Table 2. The change of ScleroID scores according to MCID values of HAQ-DI, UCLA GIT 2.0, and CHFS

	HAQ-DI			UCLA GIT 2.0			CHFS		
	Worsened (n=25)	Improved (n=22)	p	Worsened (n=34)	Improved (n=27)	p	Worsened (n=10)	Improved (n=13)	p
ΔRaynaud's	1 (5)	0 (4.8)	0.06	1 (3)	1 (3.5)	0.32	1 (1.5)	1 (3.5)	0.51
ΔHand function	**1 (6)**	**-0.5 (4)**	**0.01**	1 (2.5)	1 (3.5)	0.15	**1 (1.5)**	**-2 (3.5)**	**0.015**
ΔUpper GI	1 (3.5)	0 (3.5)	0.57	1 (3.8)	0 (3)	0.05	-1 (3.5)	0.5 (4.5)	0.97
ΔPain	**1 (5.5)**	**-1 (6)**	**0.01**	1 (6)	1 (3)	0.35	0.5 (3.5)	-0.5 (4.5)	0.42
ΔFatigue	**1 (6)**	**-2 (4.5)**	**<0.001**	0.5 (3)	0 (4)	0.08	0 (1.3)	-0.5 (3.8)	0.35
ΔLower GI	1 (7)	0 (4.8)	0.23	**2 (5)**	**-1 (3)**	**<0.001**	0.5 (6.8)	0.5 (4.5)	0.82
ΔLife choices	**2 (4.8)**	**-2 (4)**	**<0.001**	**2 (5.3)**	**0 (5.5)**	**0.008**	0.5 (4)	-1 (6.5)	0.77
ΔBody mobility	**2 (4.8)**	**-2 (4)**	**0.003**	1 (4)	0 (5)	0.07	-0.5 (3.5)	-2 (2)	0.15
ΔDyspnea	**2 (4.5)**	**0 (5)**	**0.006**	0 (5)	0 (5.3)	0.42	1 (5.3)	1 (2)	0.87
ΔDigital ulcer	1 (6)	-1.5 (7.8)	0.1	2 (6)	1 (6)	0.37	1 (6.5)	-1.5 (4.5)	0.23
ΔTotal score	**1.2 (4.1)**	**-0.6 (3.3)**	**<0.001**	**0.6 (3.4)**	**-0.3 (2.1)**	**0.03**	0.3 (2.9)	-0.4 (2.2)	0.23

HAQ-DI: Health Assessment Questionnaire Disability Index, UCLA GIT 2.0: University of California Los Angeles Scleroderma Clinical Trials Consortium gastrointestinal tract 2.0, CHFS: Cochin Hand Function Scale, GI: Gastrointestinal

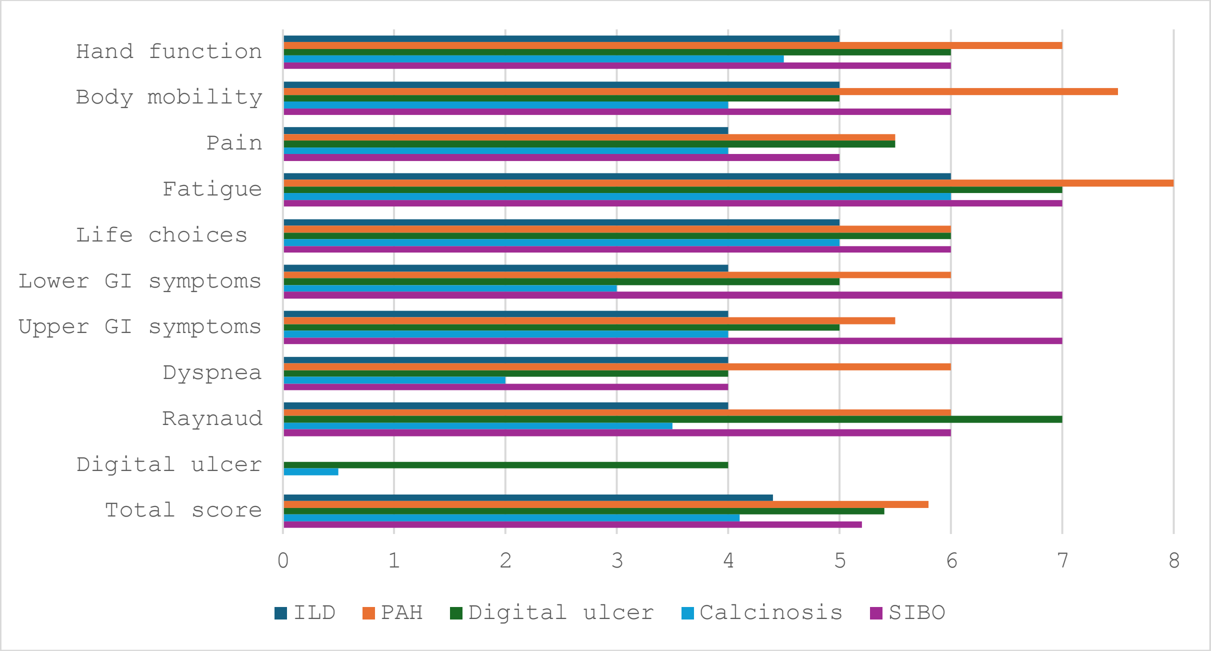

Supplementary Figure 1. Median values of the ScleroID dimensions according to main clinical manifestations. GI: Gastrointestinal, ILD: Interstitial lung disease, PAH: Pulmonary arterial hypertension, SIBO: Small intestine bacterial overgrowth

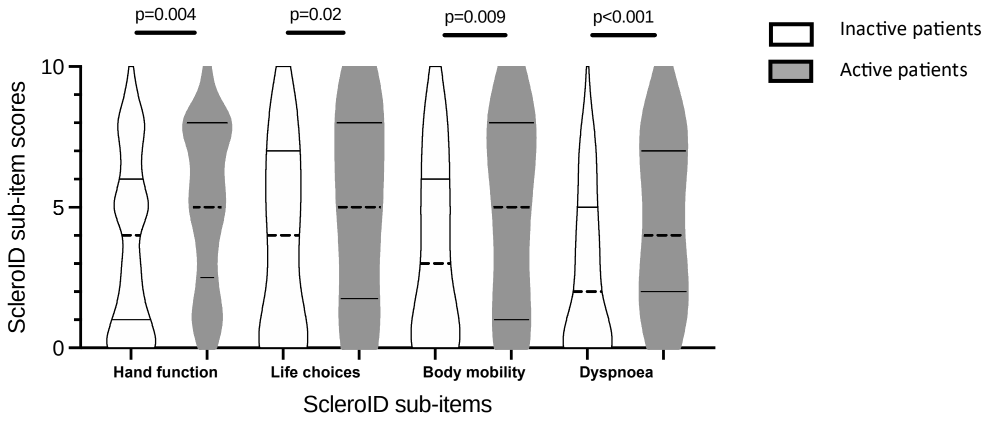

Supplementary Figure 2. Median values (dashed line) of ScleroID sub-items that had difference according to disease activity

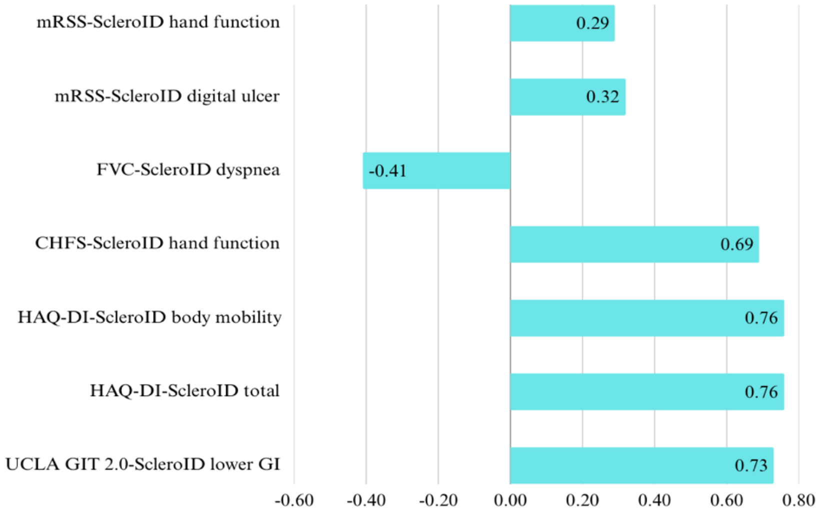

Supplementary Figure 3. A summary of the strongest correlation coefficients with main clinical findings and PROs and ScleroID sub-item and total score is shown. mRSS: Modified Rodnan skin score, FVC: Forced vital capacity, HAQ-DI: Health Assessment Questionnaire Disability Index, UCLA GIT 2.0: The University of California Los Angeles Scleroderma Clinical Trials Consortium gastrointestinal tract 2.0

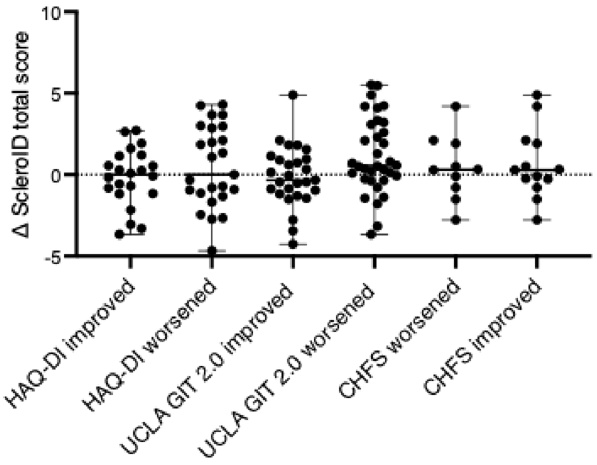

Supplementary Figure 4. The change in ScleroID total score stratified by MCID anchors. HAQ-DI: Health Assessment Questionnaire Disability Index, UCLA GIT 2.0: The University of California Los Angeles Scleroderma Clinical Trials Consortium gastrointestinal tract 2.0, CHFS: Cochin Hand Function Scale
